# Intracranial hypertension: A rare presentation of lupus nephritis

**DOI:** 10.4103/1817-1745.76111

**Published:** 2010

**Authors:** Praveen Yadav, Anishkumar Nair, Ajith Cherian, N. S. Sibi, Ashwini Kumar

**Affiliations:** Department of General Medicine, Medical College, Trivandrum, Kerala, India

**Keywords:** Anti dsDNA, anti ribosomal P protein, lupus nephritis, pseudotumor cerebri, systemic lupus erythematosus

## Abstract

A 14-year-old male presented with bilateral papilledema, growth retardation and absent secondary sexual characters. He had a past history of fever, headache and fatigue of 6 months duration. The diagnosis of intracranial hypertension (IH) was confirmed by an increased intracranial pressure and normal neuroimaging studies of the brain, except for partial empty sella, prominent perioptic cerebrospinal fluid (CSF) spaces and buckling of optic nerves. Evaluation showed erythrocyte sedimentation rate (ESR) of 150 mm/hr, positive antinuclear antibody (ANA), anti dsDNA and anti ribosomal P protein. Renal biopsy revealed diffuse segmental proliferative lupus nephritis (LN) class IV S (A) confirming the diagnosis of systemic lupus erythematosus (SLE). Treatment of LN with intravenous pulse methyl prednisolone and cyclophosphamide was effective in normalizing the CSF pressure, resulting in express and dramatic resolution of symptomatology. In a case of IH, SLE must be considered. IH, growth retardation and absence of sexual characters may be presenting manifestations of a chronic systemic inflammatory disease like SLE. These manifestations may act as a pointer to associated advanced grades of LN, which can be totally asymptomatic and missed without a renal biopsy.

## Introduction

Systemic lupus erythematosus (SLE) is an autoimmune disease characterized by diverse manifestations encompassing almost all organ systems. Neuropsychiatric lupus may range from subtle cognitive or behavioral disorders to coma and death.[1] Intracranial hypertension (IH) is included among the rare neuropsychiatric manifestations of SLE.[2–4] We discuss here a case of a 14-year-old boy who presented with features of IH and short stature, and on evaluation was found to have SLE and lupus nephritis (LN).

## Case Report

**Figure 1:**
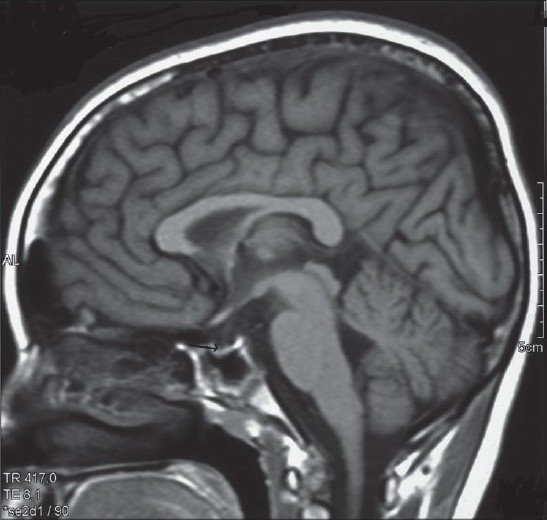
T1 sagittal magnetic resonance imaging showing reduced size of anterior pituitary gland (arrow)

A 14-year-old boy presented with 6-month duration of fever, headache and vomiting which persisted despite the use of analgesics. He was diagnosed to have hypothyroidism 4 months back for which he was on replacement thyroxine and was currently in euthyroid status. His height and weight were two standard deviations below reference. There was no history of blurring of vision, diplopia, seizures, behavioral abnormalities, arthritis, skin lesions, photosensitivity or reduction in urine output. On examination, he was febrile, had pallor, oral ulcers and absent secondary sexual characteristics. His blood pressure was normal. Neurological examination was unremarkable except for the presence of bilateral papilledema. Visual acuity was normal, but perimetry revealed bilateral concentric field constriction. Fluorescin angiography showed an increased hyperfluroscence extending beyond disc margins, confirming the diagnosis of papilledema. Magnetic resonance (MR) imaging of brain showed characteristic features of IH with partial empty sella [Figure 1], prominent perioptic cerebrospinal fluid (CSF) spaces, and buckling of optic nerves.[5] MR venogram showed no evidence of sinus thrombosis. CSF tap showed an opening pressure of 270 mm with a normal composition.

Laboratory investigations revealed microcytic hypochromic anemia, WBC count of 9110 with mild eosinophilia and erythrocyte sedimentation rate (ESR) of 150 mm/hr. Since he was an atypical patient for developing IH, he was further evaluated. Urine routine examination showed presence of albumin (+), and granular casts. Liver and renal function tests were within normal limits. Antinuclear antibody (ANA) was positive with an index of 22.7 (negative <1.4), anti dsDNA was positive at 57.67 IU/mL (negative <20 IU/mL), anti ribosomal P protein was strongly positive (+++) and anti-La antibody (SS-B) ++ positive. Serum complement levels of both C3 and C4 were low. C-reactive protein was elevated at 1.2 mg/dL (normal = 0.2–0.6 mg/dL). Anticardiolipin and lupus anticoagulant antibodies were negative. Anti neutrophil cytoplasmic antibody (both c-ANCA and p-ANCA), HbsAg, HIV, anti hepatitis C virus (anti HCV) and venereal disease research laboratory (VDRL)tests were negative. Ultrasound abdomen and echocardiography examinations were normal. Hormone estimations showed normal growth hormone, leutinizing hormone, follicle stimulating hormone and thyroid hormone levels. Fine needle aspiration cytology (FNAC) of the thyroid gland revealed lymphocytic thyroiditis. Antithyroid peroxidase antibody level was normal.

Thus, a diagnosis of SLE with IH and lymphocytic thyroiditis was made. He was treated with pulse methylprednisolone for five consecutive days. His fever disappeared, headache subsided, and papilledema decreased in the first week of treatment. Renal biopsy showed diffuse segmental proliferative LN grade IV S(A) with an activity index of 4.5/24 and a chronicity index of 0/12. He was given intravenous cyclophosphamide pulse therapy of 750 mg which was continued on monthly basis for 6 months along with daily oral steroids. At 4-week follow-up, he had become totally asymptomatic attaining clinical remission with disappearance of papilledema, and his ESR and anti dsDNA levels normalized. His steroids were subsequently tapered.

## Discussion

The syndrome of IH without structural brain or CSF abnormalities and without identifiable cause, now most appropriately termed idiopathic intracranial hypertension, was described over a century ago. While investigating the cause of IH in an atypical patient (non obese young male), we stumbled across a positive ANA and anti dsDNA titers, thus bringing the possibility of SLE in the diagnostic picture. The serum complements were reduced with a mild elevation of C-reactive protein, pointing toward a disease flare that prompted us for a renal biopsy which showed a class IV S (A) LN. He satisfied 4 out of 11 of the revised American College of Rheumatology (ACR) criteria for SLE.

IH has been described as one of the neuropsychiatric syndromes in SLE. Till date, approximately 25 adults and children manifesting IH in association with SLE have been reported.[1–4,6–8] There have been only a few reported pediatric cases in whom IH was the initial presenting sign of SLE,[4,8] as in our patient. The association of SLE and IH is still unclear. The proposed mechanisms include immune-mediated injury within the arachnoid villi and consequent reduction in CSF absorption or probable hypercoagulable state without overt vascular thrombosis giving rise to micro-obliteration of cerebral arteriolar and venous systems.[2,7,9] Steroid withdrawal in the treatment of the SLE may be a predisposing or precipitating factor in the development of IH in patients.[10] In a review of 127 patients with LN over 11 years, six patients had IH, which gave a disease prevalence of 4.7% in those with LN. Young females with serologically active lupus, severe forms of renal lesions, past history of venous or arterial thrombosis and laboratory evidences of procoagulant activity appear to be at increased risk of IH.[11]

In our patient, the antiphospholipid antibody workup was negative. Growth retardation and absence of secondary sexual characters is well described in SLE, which could be due to chronic systemic inflammatory response leading to end organ unresponsiveness (as in our patient), or associated hypopituitarism due to antiphospholipid antibody (APLA)syndrome, lymphocytic hypophysitis and chronic IH.

Anti ribosomal P protein was found to be strongly positive in our patient. It is usually associated with neuropsychiatric manifestations of SLE and LN as do high titers of anti dsDNA as seen in this case.[12]

Treatment strategy for class IV LN with intravenous pulse methyl prednisolone and cyclophosphamide was effective in normalizing the CSF pressure without the use of acetozolamide or other diuretics, giving an indirect clue that IH in SLE is immune mediated. Maintenance therapy can be achieved on oral steroids, mycophenolate mofetil or azathioprine.

In conclusion, IH can be the only presenting manifestation of SLE. Silent LN can coexist and requires enthusiastic workup, which includes a renal biopsy.

## References

[1] Quintero-Del-Rio AI, Miller V (2000). Neurologic symptoms in children with systemic lupus erythematosus. J Child Neurol.

[2] Green L, Vinker S, Amital H, Amir T, Bar-Dayan Y, Levi Y (1995). Pseudotumor cerebri in systemic lupus erythematosus. Semin Arthritis Rheum.

[3] Sbeiti S, Kayed DM, Majuri H (2003). Pseudotumor cerebri presentation of systemic lupus erythematosus: more than an association. Rheumatology.

[4] Padeh S, Passwell JH (1996). Systemic lupus erythematosus presenting as idiopathic intracranial hypertension. J Rheumatol.

[5] Hassan H, Das A, Baheti NN, Radhakrishnan A (2010). Teaching NeuroImages: idiopathic intracranial hypertension: MRI features. Neurology.

[6] Bettman JW, Daroff RB, Sanders MD, Joyt WF (1968). Papilledema and asymptomatic intracranial hypertension in systemic lupus erythematosus. A fluoresceinangiographic study of resolving papilloedema. Arch Ophthalmol.

[7] Horoshovski D, Amital H, Katz M, Shoenfeld Y (1995). Pseudotumor cerebri in SLE. Clin Rheumatol.

[8] DelGiudice GC, Scher CA, Athreya BH, Diamond GR (1986). Pseudotumor cerebri and childhood systemic lupus erythematosus. J Rheumatol.

[9] Parnass SM, Goodwin JA, Patel DV, Levinson DJ, Reinhard JD (1987). Dural sinus thrombosis: A mechanism for pseudotumor cerebri in systemic lupus erythematosus. J Rheumatol.

[10] Dave S, Longmuir R, Shah VA, Wall M, Lee AG (2008). Intracranial hypertension in systemic lupus erythematosus. Semin Ophthalmol.

[11] Nampoory MR, Johny KV, Gupta RK, Constandi JN, Nair MP, al-Muzeiri I (1997). Treatable intracranial hypertension in patients with lupus nephritis. Lupus.

[12] (2006). Renal and neuropsychiatric manifestations in patients with systemic lupus erythematosus and ribosomal P protein auto antibodies. Rev Colomb Reumatol.

